# The regulatory element READ1 epistatically influences reading and language, with both deleterious and protective alleles

**DOI:** 10.1136/jmedgenet-2015-103418

**Published:** 2015-12-12

**Authors:** Natalie R Powers, John D Eicher, Laura L Miller, Yong Kong, Shelley D Smith, Bruce F Pennington, Erik G Willcutt, Richard K Olson, Susan M Ring, Jeffrey R Gruen

**Affiliations:** 1 Investigate Medicine, Yale University, New Haven, Connecticut, USA; 2 Department of Pediatrics, Yale University, New Haven, Connecticut, USA; 3 School of Social and Community Medicine, University of Bristol, Bristol, UK; 4 Department of Molecular Biophysics and Biochemistry, Yale University, New Haven, Connecticut, USA; 5 W.M. Keck Foundation Biotechnology Resource Laboratory, Yale University, New Haven, Connecticut, USA; 6 Departments of Pediatrics and Developmental Neuroscience, University of Nebraska Medical Center, Omaha, Nebraska, USA; 7 Department of Psychology, University of Denver, Denver, Colorado, USA; 8 Institute for Behavioral Genetics, University of Colorado, Boulder, Colorado, USA; 9 Departments of Psychology and Neuroscience, University of Colorado, Boulder, Colorado, USA; 10 MRC Integrative Epidemiology Unit, University of Bristol, Bristol, UK; 11 Department of Investigative Medicine, Yale University, New Haven, Connecticut, USA

**Keywords:** dyslexia, reading disability, language impairment, Complex traits, READ1

## Abstract

**Background:**

Reading disability (RD) and language impairment (LI) are heritable learning disabilities that obstruct acquisition and use of written and spoken language, respectively. We previously reported that two risk haplotypes, each in strong linkage disequilibrium (LD) with an allele of READ1, a polymorphic compound short tandem repeat within intron 2 of risk gene *DCDC2*, are associated with RD and LI. Additionally, we showed a non-additive genetic interaction between READ1 and KIAHap, a previously reported risk haplotype in risk gene *KIAA0319*, and that READ1 binds the transcriptional regulator ETV6.

**Objective:**

To examine the hypothesis that READ1 is a transcriptional regulator of *KIAA0319*.

**Methods:**

We characterised associations between READ1 alleles and RD and LI in a large European cohort, and also assessed interactions between READ1 and KIAHap and their effect on performance on measures of reading, language and IQ. We also used family-based data to characterise the genetic interaction, and chromatin conformation capture (3C) to investigate the possibility of a physical interaction between READ1 and KIAHap.

**Results and conclusions:**

READ1 and KIAHap show interdependence—READ1 risk alleles synergise with KIAHap, whereas READ1 protective alleles act epistatically to negate the effects of KIAHap. The family data suggest that these variants interact *in trans* genetically, while the 3C results show that a region of *DCDC2* containing READ1 interacts physically with the region upstream of *KIAA0319*. These data support a model in which READ1 regulates *KIAA0319* expression through KIAHap and in which the additive effects of READ1 and KIAHap alleles are responsible for the *trans* genetic interaction.

## Introduction

Reading disability (RD) and language impairment (LI) are common, heritable learning disabilities, each involving a specific learning modality. RD, which is commonly known as dyslexia, is defined as an unexpected difficulty in processing written language in the presence of general cognitive ability that should be sufficient for proficient literacy.^1^ LI is defined as an unexpected difficulty of the same type, but with verbal language instead of written.^2^ The two disorders are closely related, involving many of the same underlying neurological processes and are frequently comorbid.^3^ ^4^ RD and LI are also highly heritable, but inheritance is complex.^3^ ^5^ Although the genetic component of both disorders has been extensively studied, few causal or functional variants have been identified. Because of the fundamental importance of language and literacy to education, affected children are often academically impeded relative to their unaffected peers, which can lead to a variety of adverse psychological, social and socioeconomic outcomes.^1^ ^3^ As RD and LI are both highly prevalent,^3^ ^4^ these adverse outcomes have an impact on society as a whole—through their cost to the health and educational systems as well as the lost potential of many affected people, whose difficulties with reading, language, or both, mask their talents and erode their confidence. RD and LI can be treated and although response to treatment varies widely, it is generally more effective at younger ages and when tailored to the individual.^2^ ^6^ A thorough understanding of the genetic components will permit better and earlier identification of individuals at risk for RD and LI and perhaps, eventually, for a priori matching of each individual to the intervention most likely to be effective.

Among the RD risk loci that have been discovered so far, the best-supported and most intriguing locus is DYX2 on chromosome 6p21.3. Several genes in this locus have been associated with RD, but two genes, *DCDC2* and *KIAA0319*, are by far the most replicated.^5^ Because these genes reside within 200 kb of each other, it was previously unknown which gene was responsible for the linkage and association of DYX2 with RD. However, emerging evidence from human, animal and cellular studies suggests that both *DCDC2* and *KIAA0319* contribute to RD.^7–18^ We recently showed that risk variants in both genes interact with each other in a non-additive manner to influence phenotype.^19^ That study, which is summarised below and which we build upon in this study, further implicated both *DCDC2* and *KIAA0319* in reading, language and IQ and identified the source of at least some of the contribution to RD and LI risk from the DYX2 locus.

In our previous study, we used a haplotype-based strategy to scan SNPs densely covering the DYX2 locus for associations with RD and LI in the Avon Longitudinal Study of Parents and Children (ALSPAC), a longitudinal birth cohort based in the former county of Avon, UK.^8^ ^20^ Using the extensive phenotypic and genetic data from approximately 5500 children of European descent in ALSPAC, we identified two six-marker risk haplotypes in the same haplotype block in *DCDC2*.^19^ One of these haplotypes was associated strongly with severe RD, the other, with severe LI. Each of these risk haplotypes was in strong linkage disequilibrium (LD) with an allele of READ1 (regulatory element associated with dyslexia 1; GenBank accession No BV677278), a compound short tandem repeat in intron 2 of *DCDC2*. READ1 is a highly polymorphic, human-specific variant, with six common alleles and 34 rare alleles described so far. A naturally occurring, 2445 bp microdeletion encompassing READ1 also exists in human populations. READ1 alleles vary primarily by the number of each of five discrete repeat units and, consequently, also vary in length. Online supplementary table S1 lists all known READ1 alleles and gives details of their structures and allele frequencies in the ALSPAC.

Our previous study added to the existing literature examining the association of READ1 and the microdeletion with RD and other related endophenotypes.^9^ ^15^ ^21–27^ Functionally, we hypothesised that READ1 serves a transcriptional regulatory role, as it specifically binds the transcriptional repressor ETV6^19^ and can modulate the activity of the *DCDC2* promoter, as shown by a luciferase reporter experiment.^15^ Because ETV6 must homodimerise to bind DNA,^28^ and because of evidence showing that it is capable of homopolymerisation,^29^ we speculated that allele structure and length—and therefore number of ETV6 binding sites—determines the regulatory power of a READ1 allele and its effect on phenotype. The two READ1 alleles in LD with the *DCDC2* risk haplotypes, alleles 5 and 6, both contain a GGAA insertion relative to the most common allele (see online supplementary table S1) and it is possible that this insertion creates a binding site for an additional ETV6 monomer.

In light of these observations, we questioned whether there might be a genetic interaction between the two *DCDC2* risk haplotypes and a known risk haplotype in *KIAA0319*, the other major RD risk gene in the DYX2 locus. The *KIAA0319* risk haplotype, which will be referred to hereafter as KIAHap for brevity, resides in a 3-marker, 77 kb haplotype block that spans approximately the 5′ half of *KIAA0319*, including its promoter, some of its upstream sequence and some of its neighbouring gene *TDP2*.^30^ KIAHap and other haplotypes and individual markers in the same 77 kb interval, have been repeatedly associated with RD, subclinical reading performance and verbal IQ.^8^ ^17^ ^31–33^ Interestingly, there is evidence that KIAHap influences *KIAA0319* expression.^16^ ^34^ We showed that individuals with at least one copy of a *DCDC2* risk haplotype *and* at least one copy of KIAHap, on average, performed worse than individuals with only one or the other (or neither), on reading, language and IQ measures.^19^ These interaction effects were greater than would be expected if the risk variants acted additively and suggested to us a regulatory interaction between READ1 and *KIAA0319*.

In order to further characterise READ1 in relation to reading and language and to examine the effects of all READ1 alleles, we genotyped and analysed READ1 by Sanger sequencing in the entire ALSPAC cohort (we had previously only genotyped READ1 in individuals with the risk haplotypes). To investigate how READ1 and KIAHap are transmitted relative to each other, we also genotyped a family-based, European-ancestry cohort from the Colorado Learning Disabilities Research Center (CLDRC). In ALSPAC, the associations of alleles 5 and 6 with severe RD and LI mirrored the associations of their respective *DCDC2* risk haplotypes in our previous study,^19^ alone and when grouped with rarer alleles of similar structure. Interestingly, another class of alleles emerged that appears both to protect against severe RD and to epistatically mask the deleterious effect of KIAHap on reading and IQ measures when present. By examining transmission of READ1 and KIAHap in the CLDRC family-based cohort, we provide circumstantial evidence that KIAHap and a given READ1 allele do not have to be *in cis* (on the same chromosome) to interact genetically. Finally, we provide evidence by chromatin conformation capture (3C) that READ1 and a region upstream of *KIAA0319* interact physically. The data reported here provide further support for the role of READ1 as a regulatory element and raise many fascinating questions about its mechanism of action.

## Methods

### Subjects, recruitment and DNA collection

Subject recruitment and collection of phenotype data and DNA for the ALSPAC cohort was completed by the ALSPAC team, as described elsewhere.^20^ A detailed description of the phenotypes and case–control criteria used in this study for ALSPAC is available in online supplementary tables S2A and S2B. The ALSPAC is a birth cohort based in the Avon region of the UK, consisting mainly of children of northern European descent, born in 1991 and 1992. Recruitment of pregnant mothers resulted in a total of 15 458 fetuses, of whom 14 701 were alive at 1 year of age. Details of the participants, recruitment and study methodologies are given in detail elsewhere.^20^ ^35^ Please note that the study website contains details of all the data that are available through a fully searchable data dictionary (http://www.bris.ac.uk/alspac/researchers/data-access/data-dictionary).

The CLDRC cohort consists of 1201 European–American individuals in 293 nuclear families. Families were recruited to the study if at least one child had a history of reading problems.^9^ ^36^

Phenotypes and exclusion criteria for this study are given in the online supplementary methods and in supplementary table S2.

### Statistical analysis

Association analysis for this study was done using SNP and Variation Suite (SVS) V.8.1.0 (Golden Helix), using a standard regression-based association test under an allelic model. A Bonferroni correction was applied to correct for multiple testing—11 tests for each phenotype. Means, SDs were obtained and an analysis of variance was performed using SPSS Statistics (IBM).

### Genotyping and 3C

Detailed methods for READ1 and SNP genotyping and the 3C experiment can be found in the online supplementary methods.

## Results

### READ1 includes both deleterious and protective alleles for RD/LI

Upon completion of READ1 genotyping in the ALSPAC, we repeated the association analysis with severe RD and severe LI previously performed with the *DCDC2* risk haplotypes. A description of the case–control definitions is given in online supplementary table S2B; they are identical to those we used in our previous study.^19^ For alleles 3, 4, 5, 6, 10 and the 2445 bp microdeletion encompassing READ1, all of which are relatively common minor alleles in Europeans (minor allele frequency (MAF) >0.035; see online supplementary table S1), we examined association with individual alleles. We also combined these with some of the rare alleles into ‘composite alleles’, in which we grouped alleles based on structural or phylogenetic similarity. For example, related alleles clustered in the same clade in a phylogenetic tree we derived previously^19^ from a ClustalW multiple alignment, under standard parameters. Online supplementary table S3 gives details of the constituents and rationales for the composite alleles. Since our previous study, the number of READ1 alleles observed has expanded from 22 to 40 (plus the microdeletion), most of which are rare.

Table 1 shows associations of READ1 with severe RD and LI. As expected, allele 5 is associated with severe RD, and allele 6 with severe LI. However, when alleles 5 and 6 are combined, the resulting composite allele is associated with both phenotypes. The same is true of ‘clade 1,’ which includes alleles 5 and 6 and rare alleles that cluster with them phylogenetically; and ‘long alleles,’ which include alleles >105 bp in length regardless of structure. By contrast, association results for another group of alleles suggest a protective effect for severe RD—they show only nominal association, but with ORs well below 1 (table 1). These alleles are denoted ‘RU1-1’ because they contain only one iteration of the 13 bp repeat unit 1 (RU1-1, the first of READ1's five repeat units), whereas most READ1 alleles contain two (see online supplementary table S3). This deletion makes RU1-1 alleles shorter than most other READ1 alleles and presumably removes some binding sites for ETV6. Allele 3 is the only common RU1-1 allele seen in Europeans (MAF=0.0456; see online supplementary table S1). The ‘short alleles’ group, which contains alleles <90 bp regardless of structure, also shows this effect, but as most of these short alleles are also RU1-1 alleles, the two categories are almost identical (see online supplementary table S3).

**Table 1:** Associations of single and composite READ1 alleles with severe RD and severe LI in the ALSPAC cohort

	Severe RD		Severe LI	
READ1 Allele	OR (95% CI)	p Value	OR (95% CI)	p Value
Allele 3	**0.47** (0.17 to 1.27)	0.0913	0.77 (0.48 to 1.23)	0.2554
Allele 4	1.24 (0.78 to 1.99)	0.3766	0.78 (0.56 to 1.09)	0.1407
Allele 5	**2.54** (1.48 to 4.36)	**0.0025926**	0.84 (0.50 to 1.40)	0.4880
Allele 6	1.54 (0.87 to 2.73)	0.1639	**1.65** (1.18 to 2.30)	0.005955*
Allele 10	0.79 (0.36 to 1.67)	0.5063	0.90 (0.59 to 1.36)	0.6034
Microdeletion	0.86 (0.48 to 1.51)	0.5810	0.85 (0.60 to 1.21)	0.3618
Alleles 5 and 6	**2.04** (1.36 to 3.08)	**0.0015725**	**1.66** (1.28 to 2.17)	**0.0003556**
Clade 1 (contains 5/6)	**1.99** (1.33 to 2.97)	**0.0020036**	**1.73** (1.34 to 2.23)	**0.00007402**
RU1-1 alleles	**0.41** (0.15 to 1.12)	0.0442*	0.80 (0.52 to 1.23)	0.2923
Short alleles	**0.41** (0.15 to 1.12)	0.0448*	0.80 (0.52 to 1.23)	0.2923
Long alleles	**2.39** (1.42 to 4.04)	**0.0033829**	**1.68** (1.17 to 2.43)	0.008962*

The association results for single and composite alleles of READ1 and the microdeletion. Values are regression-based under an allelic model. p Values that survived Bonferroni correction for multiple testing (threshold=0.05/11=0.0045) are shown in bold, with nominal associations marked with an asterisk. The highest and lowest ORs are also shown in bold. The criterion for severe RD is a score ≥2 SDs below the mean on the phoneme deletion task; the criterion for severe LI is a score of ≥2 SDs below the mean on at least one of two oral language measures (see online supplementary table S2B). For a description of the composite alleles, see online supplementary table S3, and for a detailed description of the phenotypes, see online supplementary table S2A, B.

ALSPAC, Avon Longitudinal Study of Parents and Children; LI, language impairment; RD, reading disability.

### Deleterious READ1 alleles synergise with KIAHap, whereas protective READ1 alleles epistatically negate its effect

**Figure 1:**
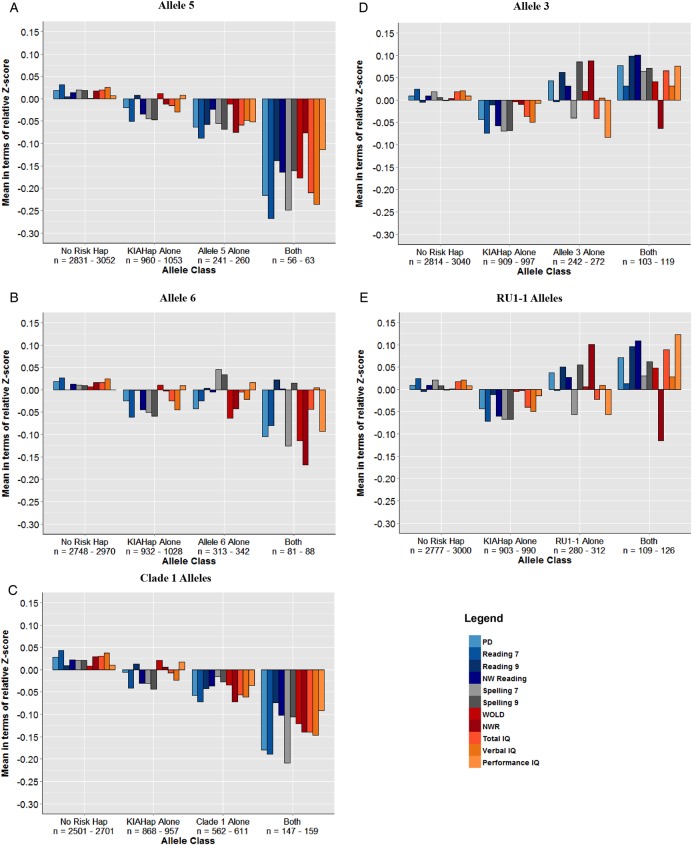
READ1–KIAHap interactions for single and composite alleles in the Avon Longitudinal Study of Parents and Children (ALSPAC). (A–E) These charts show the effect of the denoted READ1 single or composite allele on phenotype in the presence and absence of KIAHap. Each bar shows the z-score of the denoted allele class on the denoted measure, relative to the entire ALSPAC mean; units of the y-axis are fractions of a SD. Allele classes: KIAHap Alone, individuals positive for KIAHap but negative for the indicated READ1 allele; READ1 Allele Alone, individuals positive for the indicated READ1 allele but negative for KIAHap; No Risk Hap, individuals negative for both; Both, individuals positive for both. Phenotypes: PD, phoneme deletion task; Reading 7, single-word reading at age 7; Reading 9, single-word reading at age 9; NW Reading, non-word reading at age 9; Spelling 7, spelling at age 7; Spelling 9, spelling at age 9; WOLD, Wechsler Objective Language Dimensions verbal comprehension task; NWR, non-word repetition; Total, Verbal, and Performance IQ, Wechsler Intelligence Scale for Children (WISC-III). A description of these phenotypes is presented in online supplementary table S2A.

Our previous and present association results in the ALSPAC cohort prompted us to examine the effects of READ1 protective and deleterious allele classes on reading, language and IQ phenotypes in the presence and absence of KIAHap. We therefore compared mean performance on reading, language and IQ phenotypes, among individuals with different combinations of READ1 and KIAHap alleles. We performed this analysis with allele 3, allele 5, allele 6, the clade 1 alleles and the RU1-1 alleles, as these were the main classes of risk (alleles 5, 6, clade 1) and protective (allele 3, RU1-1) alleles (figure 1, table 1).

Consistent with the association results and our previous study,^19^ allele 5 interacts synergistically with KIAHap for reading phenotypes, as well as total and verbal IQ (figure 1A). Likewise, allele 6 interacts synergistically with KIAHap for non-word repetition (NWR), a common endophenotype for LI (figure 1B). WOLD (Wechsler Objective Language Dimensions), another measure used to assess LI, shows a synergistic interaction between KIAHap and both alleles 5 and 6. When alleles 5 and 6 are combined with the other rare alleles that cluster together phylogenetically into clade 1, the magnitude of the interaction is somewhat attenuated—possibly owing to the tendency of alleles 5 and 6 to associate with different phenotypes in this cohort (figure 1C). However, a one-way analysis of variance shows that mean differences between groups for the clade 1 composite allele reach statistical significance more often than do those for allele 5 or allele 6 alone (see online supplementary table S4), probably owing to the higher number of carriers and the consequent increase in statistical power.

Conversely, the effect of KIAHap for every phenotype except NWR appears to be epistatically negated in the presence of allele 3. Individuals with at least one copy of both KIAHap and allele 3, on average, perform above the population mean on all measures except NWR (figure 1D). When allele 3 is combined with the other, rare RU1-1 alleles, this trend is recapitulated for most measures (figure 1E). These interactions suggest an interdependent relationship between at least some READ1 alleles and KIAHap, where the effect of each depends on the genotype of the other.

### Transmission patterns suggest that the READ1/KIAHap genetic interaction does not occur *in cis*

Because READ1 and KIAHap reside close together on the chromosome, we questioned whether the genetic interaction was necessarily *cis*—that is, does a deleterious READ1 allele have to be on the same chromosome as KIAHap to interact genetically with it? To examine this question, we genotyped READ1 and KIAHap in the family-based CLDRC cohort and analysed transmission patterns to determine (in Europeans) how often each of the common alleles occurred *in cis* with KIAHap and how often it occurred without KIAHap. Table 2 shows the results in132 informative families (families in which at least one parent has a copy of KIAHap). Even in families selected for the presence of KIAHap, all of the common alleles occur alone more often than they occur *in cis* with KIAHap. Allele 5 is the most extreme case; out of 31 instances of allele 5 and KIAHap occurring together, we only observed one instance of the two occurring *in cis*. However, among the single deleterious READ1 alleles, allele 5 shows the strongest synergistic effect with KIAHap for reading and IQ phenotypes (figure 1). This indicates, albeit circumstantially, that READ1 and KIAHap do not need to be *in cis* to interact genetically.

**Table 2:** Linkage disequilibrium between READ1 alleles and KIAHap in the CLDRC cohort

READ1 Allele	Cis with KIAHap	Trans with KIAHap	Freq. Cis	Freq. Trans
1	199	403	0.3306	0.6694
3	33	51	0.3929	0.6071
4	33	91	0.2661	0.7339
5	1	30	0.0323	0.9677
6	10	29	0.2564	0.7436
10	7	43	0.1400	0.8600
Del	44	64	0.4074	0.5926

Transmission data for 132 informative CLDRC families (KIAHap present in at least one parent) is shown. The table shows the number of instances of each common READ1 allele (and the microdeletion) that occurred on the same chromosome as KIAHap (*cis*) versus on the other chromosome (*trans*), in all individuals carrying both that allele and KIAHap. Frequencies of *cis* and *trans* are also shown. *Cis/trans* status was elucidated by pattern of transmission in each family.

CLDRC, Colorado Learning Disabilities Research Center.

### The presence of READ1 increases intrachromosomal interactions between *DCDC2* intron 2 and the *KIAA0319* upstream region

The observations that READ1 binds a transcription factor, that KIAHap spans the promoter region of *KIAA0319* and that they appear to exhibit interdependence on each other to affect phenotype, led us to inquire whether READ1 might have a direct regulatory interaction with *KIAA0319*. To examine this question, we used 3C to determine whether READ1 and *KIAA0319* interact physically. 3C covalently crosslinks DNA and any bound proteins in their native conformation. The fixed chromatin is then fragmented, diluted and treated with DNA ligase to join fragments that are proximal to each other. If two loci interact through a transcription factor or protein complex, they would be expected to generate fusion fragments more often than would be seen by chance. Relative amounts of fusion fragments are detected by qPCR with primers designed to amplify across ligation junctions.

**Figure 2:**
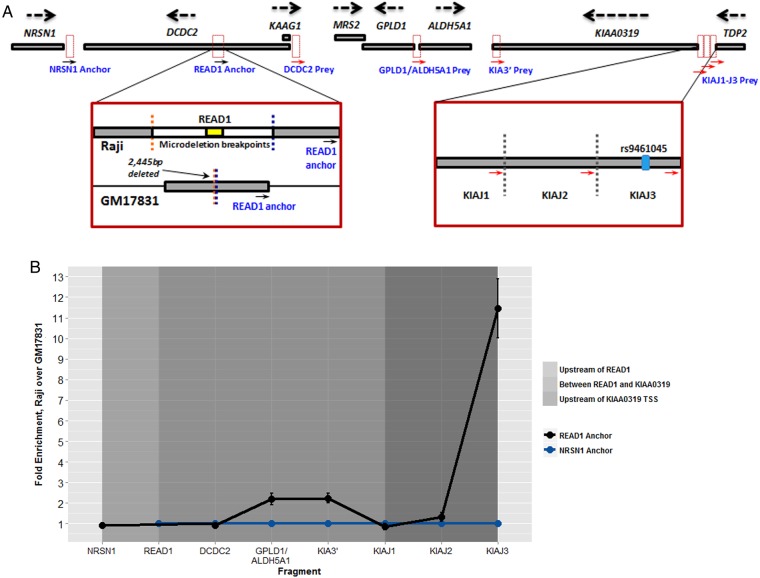
Chromatin conformation capture. (A) Schematic representation of our 3C strategy. The relevant region of the DYX2 locus is shown, with gene names in black font. Strand orientation of each gene is also shown. Dotted-line boxes show the positions of HindIII restriction fragments used for this experiment, and the positions of anchor and prey primers are indicated by arrows and labelled in blue font. Magnified views of the READ1 anchor primer and KIAJ1–J3 regions are shown. The positions of READ1, the breakpoints of the 2445 bp microdeletion (blue and orange dotted lines), and the READ1 anchor primer within the restriction fragment are shown in homozygous READ1-intact Raji cells, and GM17831 cells homozygous for the microdeletion. The primers KIAJ1–J3 flank three adjacent restriction fragments, which together encompass the intergenic region upstream of *KIAA0319* and downstream of *TDP2*. The presence of rs9461045 on the KIAJ3 fragment is noted. (B) 3C results. This graph shows enrichment of the indicated fusion fragment in Raji over GM17831, for the READ1 anchor primer relative to the control NRSN1 anchor primer. The y-axis indicates fold-enrichment of READ1-anchor fusion fragments (black line) normalised to NRSN1-anchor fusion fragments (blue line), which were set at 1. Error bars represent SE among two six-replicate qPCR experiments. Shaded areas mark the position of the included fragments relative to READ1. The prey primers shown on the x-axis are listed in the order in which they reside on the chromosome.

Figure 2A depicts our approach graphically. To assess physical interactions in the presence and absence of READ1, we chose to study two lymphoblastoid cell lines—GM17831, which is homozygous for the 2445 bp microdeletion encompassing READ1; and Raji, which is homozygous intact for this 2445 bp region. Raji cells also contain a READ1 risk allele; the READ1 genotype of Raji cells is 4,5. We chose HindIII as the restriction enzyme because it generates a restriction fragment containing the entire 2445 bp microdeletion interval; the flanking HindIII sites are therefore still present in a cell line homozygous for the microdeletion. HindIII also generates three restriction fragments in and around the *KIAA0319* promoter (figure 2A). We used two anchor primers for this experiment: one flanking the HindIII site on the READ1 restriction fragment, the other flanking the HindIII site on a restriction fragment near the *NRSN1* promoter, outside any loop that would occur between READ1 and *KIAA0319*, as a control. Prey primers flank the three HindIII fragments near the *KIAA0319* transcription start site (KIAJ1, KIAJ2, KIAJ3), the region upstream of *DCDC2* (DCDC2), the region upstream of both *GPLD1* and *ALDH5A1* and the *KIAA0319* 3′ untranslated region (KIA3′) (figure 2A).

Figure 2B shows the combined results of two six-replicate qPCR experiments (12 experiments in all). We first calculated fusion fragment enrichment in Raji over GM17831, corrected for digestion efficiency and normalised to a control amplicon (ACTβ) that does not contain a HindIII site. For each prey primer, we then compared these values between the READ1 and NRSN1 anchor primers. If READ1 does not interact specifically with a given region of DYX2, there should be no difference in Raji/GM17831 enrichment between the anchor primers for the corresponding prey primer. That is, the presence (Raji) or absence (GM17831) of READ1 should not make a difference if it does not physically interact with that region of the locus. As shown in the figure, there is no difference between the READ1 and NRSN1 anchor primers upstream of the *DCDC2* promoter, or at KIAJ1 or KIAJ2, but a small difference is apparent in the region between the *DCDC2* and KIAJ1 fragments, which disappears at KIAJ1 and KIAJ2, then reappears much more strongly at KIAJ3. Interestingly, the KIAJ3 fragment contains rs9461045, a SNP previously proposed to be a functional variant in KIAHap and shown to cause an allelic reduction in *KIAA0319* expression in several cell lines.^16^ These results suggest that READ1 increases the probability of this interaction when it is present in comparison with when it is absent and probably indicates a direct regulatory interaction between READ1 and the *KIAA0319* gene. They may also suggest that READ1 interacts with (and may regulate) other genes in the locus, including *GPLD1* and *ALDH5A1*, albeit much less strongly.

## Discussion

In previous work, we provided strong evidence that READ1 is a transcriptional regulatory element that interacts non-additively with KIAHap, a risk haplotype spanning the 5′ half of *KIAA0319*.^19^ This evidence, though compelling, was indirect; the variants associating with RD and LI and interacting with KIAHap were not alleles of READ1 itself, but two six-SNP haplotypes in strong LD with two alleles of READ1. In this further study, we were able to examine the effects of all READ1 alleles in the large, ethnically homogeneous ALSPAC cohort. The results suggest at least two classes of READ1 alleles in European populations: deleterious and protective. The association results show this for severe RD, while it is suggested by genetic interaction analysis for most of the reading, language and IQ phenotypes considered in this study. This indicates that READ1 is a functional variant in the region and provides insight into its mechanism of action. First and foremost, whether an allele is deleterious or protective seems to depend on its length and/or structure; longer alleles with insertions in repeat unit 2 tend to be deleterious, while shorter alleles with a deletion of one copy of repeat unit 1 tend to be protective. As repeat unit 1 was the major *in vitro* ETV6 binding probe in our previous electrophoretic mobility shift assay (EMSA) and stable isotope labelling by amino acids in cell culture (SILAC) experiments,^19^ this is consistent with our model that indels in READ1 change the size of the ETV6 homopolymer that can bind and thus alter the regulatory power of the allele.

Interestingly, the genetic interaction between READ1 and KIAHap is different for different classes of READ1 alleles. Deleterious READ1 alleles synergise with KIAHap to reduce performance on reading, language and IQ measures more than would be expected if these READ1 alleles and KIAHap acted additively. By contrast, protective READ1 alleles epistatically suppress the deleterious effect of KIAHap: performance on reading-related measures is typically at or above the population mean in the RU1-1-positive group, regardless of the presence or absence of KIAHap. Although this increase in performance is slight, it shows that the small deleterious population effect of KIAHap on reading performance does not occur when RU1-1 is present. In other words, for reading-related measures, KIAHap does not confer risk for poorer performance in the presence of an RU1-1 allele. Similarly, the deleterious READ1 alleles alone, like KIAHap alone, reduce mean performance only slightly, whereas their effects are greater in the presence of each other. This apparent genetic interdependence lends a contextual dimension to these ‘risk variants’: if used in the clinic to assess individual risk, they cannot be considered apart from each other.

Although the READ1–KIAHap genetic interaction shows strong general trends, there is some variability among phenotypes. For instance, single-word reading shows a somewhat attenuated effect at age 9 (reading 9) versus at age 7 (reading 7) (figure 1). This may be due to the measures themselves: ALSPAC's reading task at age 9 is abbreviated compared with that at age 7 and therefore may not capture reading ability with the same resolution. However, the effect of instruction is also likely to be important. At age 7, formal reading instruction is in many cases just beginning, while at age 9, the quality of instruction is expected to exert significantly greater influence on reading performance.^37^ A stronger genetic effect at age 7 would be expected. There is also some disparity between the two language measures: NWR and verbal comprehension (WOLD). This is not unexpected, as these tasks measure different aspects of verbal language. NWR, in which the child listens to a non-word and repeats it to the examiner, measures receptive phonological working memory, as well as other language skills such as phonological processing and articulation.^38^ WOLD, in which the child answers questions about a story read to him/her by the examiner, measures ability to derive meaning from spoken language.^39^ This variability in the effect of the READ1–KIAHap genetic interaction points to the complex nature of reading, language and cognitive traits presented here.

Several independent lines of evidence point to a direct regulatory interaction between READ1 and *KIAA0319*, including their genetic interaction, the different effects of structurally distinct alleles on this interaction, the binding of the potent transcriptional repressor ETV6 to READ1 and now, the physical interaction between READ1 and a promoter-proximal region of *KIAA0319* shown by 3C. More specifically, these data show higher fusion fragment enrichment in Raji (a homozygous READ1-intact lymphoblastoid cell line that contains a risk allele of READ1) relative to GM17831 (a homozygous READ1-deleted lymphoblastoid cell line) for the READ1 anchor primer versus the control NRSN1 anchor primer. This enrichment is also present for *GPLD1* and KIA3′—two fragments in the region between *DCDC2* and KIAJ1—but decays at the KIAJ1 and KIAJ2 fragments and then reappears very strongly at KIAJ3 (figure 2B). Intriguingly, we did not observe an interaction between READ1 and fragments containing the *KIAA0319* promoter (KIAJ1) or the *DCDC2* promoter (DCDC2). This appears to indicate that the functional variant responsible for the READ1–KIAHap genetic interaction is located upstream of *KIAA0319*. This region has been previously shown to affect *KIAA0319* expression by implication of an RD-associated allele of SNP rs9461045 with reduced *KIAA0319* expression in several cell lines.^16^

**Figure 3:**
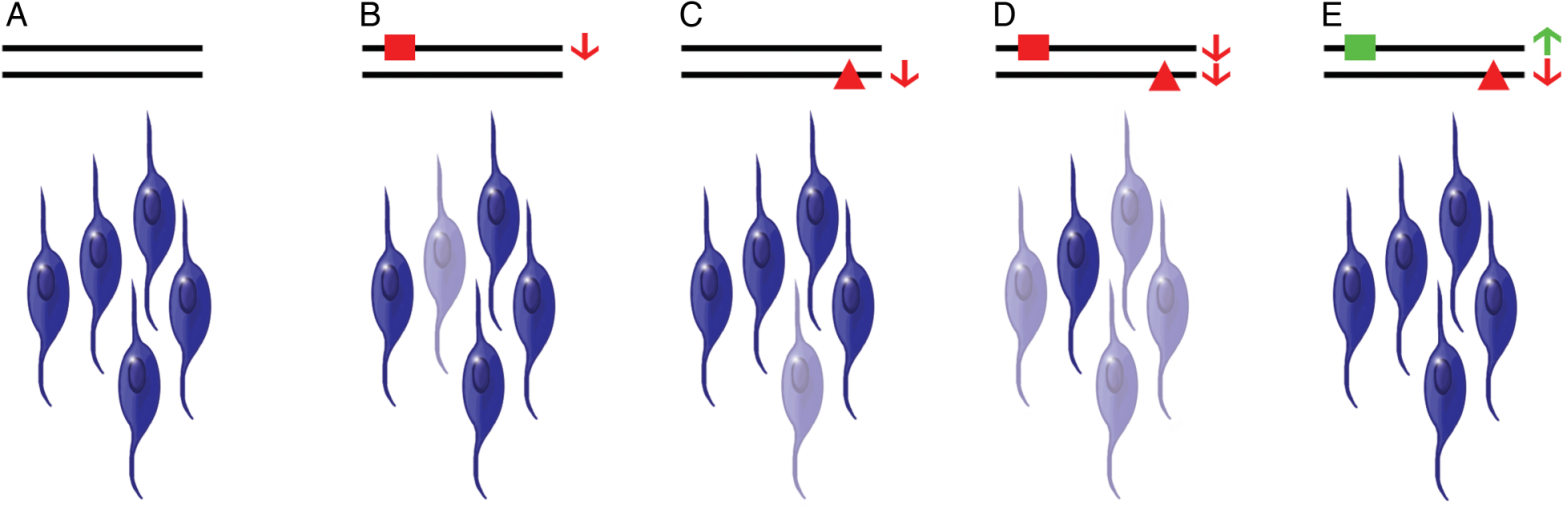
Theoretical model of the READ1–KIAHap genetic interaction. (A) In the presence of neutral READ1 alleles and the absence of KIAHap, most neural progenitor cells/neurons express *KIAA0319* above a minimal threshold (non-faded cells). (B and C) In the presence of a deleterious READ1 allele (red square) or KIAHap alone (red triangle), *KIAA0319* from the affected allele decreases, dropping overall *KIAA0319* expression in some cells below the minimal threshold (faded cells), slightly increasing the probability of problems with reading and language. (D) In the presence of both a deleterious READ1 allele and KIAHap *in trans*, *KIAA0319* expression from both alleles decreases, dropping overall *KIAA0319* expression below the minimal threshold in many cells, substantially increasing the probability of problems with reading and language. (E) A compensatory increase in *KIAA0319* expression due to a protective READ1 allele (green square) negates the decrease in expression due to KIAHap, maintaining overall *KIAA0319* expression above the minimal threshold in this case.

Although the genetic interaction between READ1 and KIAHap is clear, it is interesting that it is not necessarily a *cis* interaction. The transmission data in the CLDRC cohort show that allele 5, which synergises strongly with KIAHap for reading and IQ measures, rarely occurs on the same chromosome as KIAHap in Europeans (table 2). When considered with the 3C results, this creates an apparent paradox: the two variants interact *in cis* physically, but *in trans* genetically. Transvection would resolve this paradox, but while we cannot definitively rule it out, we consider this possibility unlikely because homologue pairing in somatic cells is strictly limited in mammals.^40^ Our model, illustrated in figure 3, resolves the paradox in terms of total *KIAA0319* expression from both chromosomes. Under this model, READ1 regulates *KIAA0319* gene expression *in cis*—that is, each READ1 allele directly regulates only the copy of *KIAA0319* on the same chromosome as itself. KIAHap likewise alters *KIAA0319* expression *in cis*. If deleterious READ1 alleles and KIAHap do indeed decrease expression of *KIAA0319*, the *additive* effect of these deleterious variants could drop average *KIAA0319* expression below a tolerable threshold. If enough cells (eg, neurons or neural progenitors) express *KIAA0319* below this threshold, inadequate KIAA0319 will be elaborated, increasing the risk for reading and verbal language problems. Under our model (figure 3), the presence of both a READ1 risk variant and KIAHap would drop *KIAA0319* expression below this threshold in many more cells than the presence of only one or the other. The mechanism by which *KIAA0319* expression influences reading and language is unknown, but KIAA0319 is thought to be a signalling protein and is known to be involved in neuronal migration and dendrite outgrowth.^10^ ^41^

The model explains the epistatic effect of the RU1-1 alleles over KIAHap the same way. These alleles, which have lost some ETV6 binding sites, may have lost enough of their repressive power to allow *KIAA0319* expression to be relatively higher, thereby compensating for reductions in expression caused by deleterious READ1 alleles and/or KIAHap. This model also allows for considerable phenotypic variation among individuals with the same genotype, as gene expression in individual cells can be influenced by many genetic, epigenetic, environmental and stochastic factors. In spite of this complexity, READ1 and KIAHap have a clear effect on population risk of RD in Europeans and may be useful in assessing individual risk if included in a model with environmental risk factors and other genetic risk variants.

Another perplexing facet of this interaction is that the long allele–deleterious/short allele–protective trend, while compelling, is certainly not the whole story. The RU1/RU2 region is also only part of the puzzle, as shown by the different effects of allele 5 and allele 6 on phenotype. These alleles differ by only 4 bp in RU4 (see online supplementary table S1), yet allele 5 has a stronger effect than allele 6, and also preferentially affects reading-related and IQ measures, whereas allele 6 mainly affects verbal language (figure 1A, B). When the two alleles are combined together and with the other, rare clade 1 alleles (figure 1C), the magnitude of their synergistic effects appears somewhat attenuated, suggesting that alleles 5 and 6 are the main drivers for their respective phenotypes.

Taken together, the results presented here broadly suggest a model in which READ1 alleles differentially suppress *KIAA0319* expression through a direct, *cis*-regulatory interaction, the magnitude of which depends on the structure of the READ1 allele, and also on the presence or absence of a variant in LD with KIAHap, possibly rs9461045. Under our model, the additive effects on *KIAA0319* gene expression of READ1 and KIAHap genotypes on the two homologous chromosomes are responsible for the apparent *trans* genetic interaction. Physical interaction between READ1 and the *KIAA0319* upstream region appears to be restricted to the KIAJ3 restriction fragment, but there are interactions with other regions of the locus too, including the upstream regions of *GPLD1/ALDH5A1*, but surprisingly not *DCDC2*. This may imply that READ1 can regulate other genes in the locus and that its preference for its binding site upstream of *KIAA0319* can be altered by variants in these regions.

This study confirms and elaborates our previous work, but also raises many tantalising questions about the READ1–*KIAA0319* interaction. For instance, what other genetic and environmental factors can influence this interaction? Exactly what effect do these variants and their interaction have on neural development and how do they exert it? Much further work will be required to answer these and other questions, but the answers will provide a case of interacting regulatory variants that influence highly heritable complex traits in humans—a model that may well be broadly applicable to complex inheritance.

## Supplementary Material

Web supplement
