# Supplementary material for: The regulatory element READ1 epistatically influences reading and language, with both deleterious and protective alleles
Source: J Med Genet. 2015 Dec 12;53(3):163–71. doi: 10.1136/jmedgenet-2015-103418 (PMC4789805; doi:10.1136/jmedgenet-2015-103418)
Supplement: Web supplement [file jmedgenet-2015-103418-s1.pdf]

Supplementary Material

The Regulatory Element READ1 Epistatically Influences Reading and Language, with both Deleterious and Protective Alleles

Authors: Natalie R. Powers[1,2,], John D. Eicher[1,], Laura L. Miller[3], Yong Kong[4,5], Shelley D. Smith[6], Bruce F. Pennington[7], Erik G. Willcutt[8,9], Richard K. Olson[8,9], Susan M. Ring[3,10], Jeffrey R. Gruen[1,2,11,*]

*Corresponding author
E-mail: jeffrey.gruen@yale.edu
Telephone: (203) 737-2202
Fax: (203) 737-5972

Supplementary Methods

Phenotypes and Exclusion Criteria

The children of the ALSPAC have been extensively phenotyped from before birth to early adulthood. An update on the status of the cohort was published recently. [1] The reading, language, and cognitive measures used for this study were collected at ages 7, 8, and 9 years. DNA samples from 10,259 of these children were available for genotyping. Because this is a follow-up of our previous study, for the ALSPAC we used the same dataset and the same case/control definitions for severe RD and LI as reported in our previous study. Briefly, subjects were excluded if they scored below 75 on the WISC-III full-scale IQ measure, if they reported an ancestry other than European, or if their DNA samples did not meet a minimum standard of quality. Subjects were also excluded if they did not have an unequivocal READ1 genotype, or if phasing of KIAHap returned a posterior probability value below 0.95. After all exclusion criteria were applied, a final ALSPAC dataset of 4,428 subjects remained for analysis. Details of the phenotypes and case/control definitions for ALSPAC are presented in Supplementary Table 2A-B. The Colorado Learning Disabilities Research Center (CLDRC) cohort consists of families with twin pairs and their siblings; families were recruited to the study if at least one member of each twin pair had a history of reading problems. For this study, 1,188 individuals in 292 families were used for analysis, after exclusion of several families that showed Mendelian errors in a SNP dataset we used previously. In the case of monozygotic twins, only one member of each twin pair was used for transmission analysis in this study. A full description of the cohort and its phenotypic measures is available in Gayán et al. (1999). [2] For transmission assessment in the CLDRC, families were only included if at least one copy of KIAHap was present in at least one of the parents.

READ1 Genotyping

READ1 and the 2,445bp microdeletion encompassing it were genotyped by Sanger sequencing and allele-specific PCR, respectively. Primers and amplification protocols are described in detail elsewhere. [3] For microdeletion genotyping, PCR products were electrophoresed on large (150-250 well) 1% agarose gels at 150V for approximately 1.5h. Gels were imaged and documented with a Kodak 1D documentation system, and genotypes were called manually from the gels. For READ1 genotyping, purification of PCR products and Sanger sequencing were done by the Keck DNA Sequencing Lab at Yale University, according to standard protocols. Genotypes were called from the chromatograms using an in-house C++ program developed by YK (available upon request). In cases where the program detected errors, chromatograms were read and de-convoluted manually; such errors often yielded a new allele. Call rates in ALSPAC for READ1 and the microdeletion were 0.963 and 0.993, respectively.

SNP Genotyping

rs4504469, rs2038137, and rs2143340 were genotyped in ALSPAC by Scerri et al., as described. [4] Call rates for these three SNPs in our dataset were 0.891 0.900 and 0.896, respectively. For the CLDRC sample, the SNPs rs4504469, rs2038137, and rs2143340 were genotyped at the University of Nebraska Medical Center as part of an Illumina BeadXpress array. Parents and siblings were genotyped, and the calls were reviewed for genotyping quality and for Mendelian inconsistencies. Samples or SNPs with poor performance were excluded from further analysis. After cleaning, the call rates were 0.987, 0.960, and 0.993, respectively. Phasing of these SNPs to construct individual haplotypes was done using the –hap-phase function in PLINK. Only samples with high-confidence phasing (posterior probability greater than or equal to 0.95) were used for analysis.

Cell Culture

Raji cells (ATCC CCL-86) were grown at 37ºC, 5% CO_2, in RPMI 1640 medium supplemented with 10% FBS. GM17831 cells (Coriell) were grown under the same conditions in RPMI 1640 medium supplemented with 15% FBS.

Chromatin Conformation Capture (3C)

3C was done as previously described, [5] with several minor modifications. For each 3C prep, 100 million freshly harvested cells were fixed in 1% formaldehyde in 21mL PBS, at room temperature for 10min. Formaldehyde was then quenched for 5 min at room temperature after addition of 1.26mL of 2.5M glycine. After fixation, cells were pelleted, flash-frozen in liquid nitrogen, and stored at -80ºC until used for 3C. For 3C, cells were resuspended in 6 volumes of Lysis buffer 1 (10mM Tris-HCl ph=8.0, 10mM NaCl, 0.2% Igepal), and homogenized with 7 strokes in a Dounce homogenizer. They were then incubated on ice for 20min, and homogenized

again with 25 strokes in a Dounce homogenizer. The nuclei were collected by centrifugation (5min, 2500g, 4ºC), washed with 500ul 1X restriction Buffer 2.1 (NEB), collected by centrifugation (same settings), resuspended in 1X Buffer 2.1, and split into 10 aliquots, ~60µl each. To each aliquot, 312µl Buffer 2.1 and 38µl 1% SDS was added, and the aliquots were incubated with rotation at 65ºC for 15min. 44µl 10% Triton X-100 were added to each tube, and 400 units of HindIII restriction enzyme (NEB) were added to 9 of the tubes. The remaining tube was split into two aliquots, and 200 units of HindIII were added to one of them (digested control); the other tube served as an undigested control. All tubes were incubated overnight at 37ºC with rotation. The following morning, an additional 200 units of HindIII were added to the 9 3C digests, and an additional 100 units to the digested control, and the tubes were incubated at 37ºC for an additional 2 hours with rotation. The enzyme was then inactivated by addition of 43µl of 20% SDS to each of the 9 3C digests, and 20µl each to the digested and undigested controls, and incubating at 65ºC with rotation for 30 minutes. Ligation reactions were then set up. Each of the 9 reactions consisted of 1 3C digest, 745µl 10X ligation buffer (500mM Tris-HCl, pH=7.5, 100mM $MgCl_2$, and 100mM dithiothreitol), 745µl 10% Triton X-100, 8µl BSA (100mg/mL), 1µl T4 DNA ligase (30 Weiss units/µl), and 5.5mL H_2O. The reactions were mixed and allowed to proceed at 16ºC for 3 hours. Meanwhile, the digested and undigested controls were treated with 10µg RNAse A and incubated for 1 hour at 37ºC. When ligation was complete, the ligation reactions were treated with 50µl proteinase K (25mg/mL), and incubated overnight at 65ºC to reverse crosslinks and digest protein. The digested and undigested controls were subjected to the same treatment, with 10µl proteinase K. The following morning, an additional 25µl and an additional 5µl of proteinase K was added to each ligation reaction and to each of the controls, respectively; they were then incubated at 65ºC for an additional 2 hours. The digested and undigested controls were stored at -20ºC until further use. The ligation reactions were distributed among 11 MaxTract tubes (Qiagen) for DNA purification. An equal volume of phenol-chloroform-isoamyl alcohol (25:24:1) was added to each tube. The tubes were inverted several times, and spun 5 minutes at 1,500xg. The aqueous phases were decanted and combined into 4 30mL glass centrifuge tubes. To the solution in each tube, 0.7vol isopropanol and 0.1vol 3M sodium acetate (pH=5.2) was added, the tubes were mixed by inversion, and placed at -80ºC for 1 hour. They were the thawed at room temperature for 30min, and spun for 45 minutes at 14,000xg (4ºC). The supernatant was decanted, the pellets were dried at room temperature, and each was dissolved in 250µl 1X TE (10mM Tris-HCl, pH=8.0, 1mM EDTA). The DNA was combined into 1mL total, transferred to a 1.5mL tube, and treated with 100µg RNAse A. The RNAse reaction was allowed to proceed at 37ºC for 1 hour. The DNA was then split into 2 500µl aliquots, and each was added to a 2mL phase-lock tube. The digested and undigested controls were each also added to a phase-lock tube. An equal volume of phenol-chloroform-isoamyl alcohol (25:24:1) was added to each tube, the tubes were inverted to mix, and spun 5min at 16,000xg (room temperature). The same volume of chloroform was added to the aqueous phase of each tube, and the tubes were again inverted to mix and spun at 16,000xg for 5min. The aqueous phase was then collected and transferred to a fresh 2mL tube, and 0.1vol of 3M sodium

acetate (pH=5.2) and 2.5vol of ice cold 100% ethanol was added to each tube. The tubes were inverted to mix, and stored at -80ºC overnight to precipitate the DNA. The following morning, the tubes were spun for 45min, 16,000xg, at 4ºC. The 3C pellets were washed 5X with cold 70% ethanol; the digested and undigested controls, 2X. The pellets were then dried, and each 3C pellet was dissolved in 500µl 1X TE, while the digested and undigested control pellets were dissolved in 150µl 1X TE. The two dissolved 3C pellets were combined into one, and all three samples were quantified with PicoGreen (Life Technologies), as per manufacturer's instructions. Samples were then used as qPCR template. The digested and undigested controls were used to correct for digestion efficiency between the two cell lines, as previously described. [5]

qPCR

qPCR was done with the QuantiTect SYBRGreen qPCR kit from Qiagen, in 50µl reactions, as per manufacturer's instructions. Primers are listed in Supplementary Table 5. The qPCR reaction is as follows: 15 min at 95ºC, then 45 cycles of 30 sec at 95ºC followed by 30 sec at 60ºC followed by 1 min at 72ºC, then 6 min at 72ºC, and an indefinite hold at 4ºC. For qPCR reactions, 3C template DNA was diluted to a final concentration of 20ng/ul, and each primer was diluted to a final concentration of 0.25µM. qPCR results were normalized across templates to a control amplicon from the gene encoding β-actin (*ACTβ*). The *ACTβ* primers amplify across a region without a HindIII, BamH1, or BglII site.

β-globin Control Experiment

To assess the effectiveness of our 3C protocol, and to eliminate any systematic differences between Raji and GM17831 cells, we performed 3C, according to the above protocol, with a set of previously described intrachromosomal interactions in the β-globin locus. Vu et al. (2010) detail two interactions and one non-interaction with an LCR region in the locus (flanked by anchor primer C). [6] One is a strong local interaction with a nearby region (flanked by prey primer B), one is a weaker long-range interaction (flanked by prey primer A), and one is a non-interaction with a distant region (flanked by prey primer D). Globin primer sequences are listed in Supplementary Table 6. Because the globin primers flank BglII or BamH1 sites rather than HindIII sites, fixed cells were subjected to double-digests with these enzymes in NEB restriction buffer 3.1. For each 3C digest, 200 units of each enzyme were used (100 units of each for the digested control). All other aspects of the protocol are unchanged, except an annealing temperature of 65ºC rather than 60ºC was used for qPCR with the globin primers. The results of the control experiment are shown in Supplementary Figure 1. They agree with those of Vu et al. (2010), [6] and show no difference in enrichment between Raji and GM17831 cells at the β-globin locus—indicating an effective 3C protocol and suggesting that there is no systematic bias between the two cell lines.

Supplementary Tables and Figures

Allele	Repeat unit 1	Repeat unit 2	SNP1	Repeat unit 3	Const. Region	Repeat unit 4	Repeat Unit 5	Length (bp)	Allele Frequency
**1**	**(GAGAGGAAGGAAA)2**	**(GGAA)7**	**(GAAA)1**	**(GGAA)2**	**GGAAAGAATGAA**	**(GGAA)4**	**(GGGA)2**	**102**	**0.6286**
2	(GAGAGGAAGGAAA)1	(GGAA)9	(GAAA)0	(GGAA)0	GGAAAGAATGAA	(GGAA)4	(GGGA)2	85	0
**3**	**(GAGAGGAAGGAAA)1**	**(GGAA)6**	**(GAAA)1**	**(GGAA)2**	**GGAAAGAATGAA**	**(GGAA)4**	**(GGGA)2**	**85**	**0.0456**
**4**	**(GAGAGGAAGGAAA)2**	**(GGAA)6**	**(GAAA)1**	**(GGAA)2**	**GGAAAGAATGAA**	**(GGAA)4**	**(GGGA)2**	**98**	**0.0955**
**5**	**(GAGAGGAAGGAAA)2**	**(GGAA)8**	**(GAAA)1**	**(GGAA)2**	**GGAAAGAATGAA**	**(GGAA)4**	**(GGGA)2**	**106**	**0.0361**
**6**	**(GAGAGGAAGGAAA)2**	**(GGAA)8**	**(GAAA)1**	**(GGAA)2**	**GGAAAGAATGAA**	**(GGAA)3**	**(GGGA)2**	**102**	**0.0471**
7	(GAGAGGAAGGAAA)2	(GGAA)8	(GAAA)1	(GGAA)1	GGAAAGAATGAA	(GGAA)4	(GGGA)2	102	0
8	(GAGAGGAAGGAAA)2	(GGAA)7	(GAAA)0	(GGAA)0	GGAAAGAATGAA	(GGAA)4	(GGGA)2	90	$5.136x10^{-5}$
9	(GAGAGGAAGGAAA)1	(GGAA)7	(GAAA)1	(GGAA)2	GGAAAGAATGAA	(GGAA)4	(GGGA)2	89	0.0063
**10**	**(GAGAGGAAGGAAA)2**	**(GGAA)4**	**(GAAA)1**	**(GGAA)2**	**GGAAAGAATGAA**	**(GGAA)4**	**(GGGA)2**	**90**	**0.0467**
11	(GAGAGGAAGGAAA)2	(GGAA)7	(GAAA)1	(GGAA)2	GGAAAGAATGAA	(GGAA)3	(GGGA)2	98	0.0014
12	(GAGAGGAAGGAAA)1	(GGAA)8	(GAAA)1	(GGAA)2	GGAAAGAATGAA	(GGAA)3	(GGGA)2	89	$5.138x10^{-5}$
13	(GAGAGGAAGGAAA)2	(GGAA)9	(GAAA)1	(GGAA)2	GGAAAGAATGAA	(GGAA)3	(GGGA)2	106	0.0012
14	(GAGAGGAAGGAAA)2	(GGAA)9	(GAAA)1	(GGAA)2	GGAAAGAATGAA	(GGAA)4	(GGGA)2	110	0.0016
15	(GAGAGGAAGGAAA)2	(GGAA)5	(GAAA)2	(GGAA)2	GGAAAGAATGAA	(GGAA)4	(GGGA)2	98	0.0005
16	(GAGAGGAAGGAAA)2	(GGAA)5	(GAAA)1	(GGAA)2	GGAAAGAATGAA	(GGAA)4	(GGGA)2	94	0.0015
17	(GAGAGGAAGGAAA)2	(GGAA)4	(GAAA)2	(GGAA)2	GGAAAGAATGAA	(GGAA)4	(GGGA)2	94	0
18	(GAGAGGAAGGAAA)2	(GGAA)7	(GAAA)2	(GGAA)2	GGAAAGAATGAA	(GGAA)4	(GGGA)2	106	0.0003
19	(GAGAGGAAGGAAA)2	(GGAA)9	(GAAA)0	(GGAA)0	GGAAAGAATGAA	(GGAA)4	(GGGA)2	98	$5.138x10^{-5}$
20	(GAGAGGAAGGAAA)2	(GGAA)10	(GAAA)1	(GGAA)2	GGAAAGAATGAA	(GGAA)4	(GGGA)2	114	0.0004
21	(GAGAGGAAGGAAA)2	(GGAA)6	(GAAA)1	(GGAA)2	GGAAAGAATGAA	(GGAA)3	(GGGA)2	94	$5.138x10^{-5}$
22	(GAGAGGAAGGAAA)2	(GGAA)10	(GAAA)0	(GGAA)0	GGAAAGAATGAA	(GGAA)4	(GGGA)2	102	0.0027
23	(GAGAGGAAGGAAA)2	(GGAA)11	(GAAA)0	(GGAA)0	GGAAAGAATGAA	(GGAA)4	(GGGA)2	106	0.0001
24	(GAGAGGAAGGAAA)2	(GGAA)6	(GAAA)2	(GGAA)2	GGAAAGAATGAA	(GGAA)4	(GGGA)2	102	0.0002
25	(GAGAGGAAGGAAA)1	(GGAA)8	(GAAA)1	(GGAA)2	GGAAAGAATGAA	(GGAA)4	(GGGA)2	93	$5.138x10^{-5}$
26	(GAGAGGAAGGAAA)2	(GGAA)5	(GAAA)1	(GGAA)2	GGAAAGAATGAA	(GGAA)3	(GGGA)2	90	0.0001
27	(GAGAGGAAGGAAA)1	(GGAA)5	(GAAA)1	(GGAA)2	GGAAAGAATGAA	(GGAA)4	(GGGA)2	81	0.0001
28	(GAGAGGAAGGAAA)2	(GGAA)7	(GAAA)1	(GGAA)2	GGAAAGAATGAA	(GGAA)5	(GGGA)2	106	0.0002
29	(GAGAGGAAGGAAA)2	(GGAA)5+(GGGA)1+(GGAA)1	(GAAA)1	(GGAA)2	GGAAAGAATGAA	(GGAA)4	(GGGA)2	102	$5.138x10^{-5}$

30	(GAGAGGAAGGAAA)2	(GGAA)5	(GAAA)1	(GGAA)4	GGAAAGAATGAA	(GGAA)4	(GGGA)2	102	$5.138x10^{-5}$
31	(GAGAGGAAGGAAA)2	(GGAA)7	(GAAA)1	(GGAA)1+ (GGGA)1	GGAAAGAATGAA	(GGAA)4	(GGGA)2	102	$5.138x10^{-5}$
32	(GAGAGGAAGGAAA)2	(GGAA)8	(GAAA)0	(GGAA)0	GGAAAGAATGAA	(GGAA)4	(GGGA)2	94	0.0001
33	(GAGAGGAAGGAAA)2	(GGAA)6	(GAAA)0	(GGAA)0	GGAAAGAATGAA	(GGAA)3	(GGGA)2	82	$5.138x10^{-5}$
34	(GAGAGGAAGGAAA)2	(GGAA)7	(GAAA)2	(GGAA)2	GGAAAGAATGAA	(GGAA)3	(GGGA)2	102	$5.138x10^{-5}$
35	(GAGAGGAAGGAAA)1+(GAGAGGAAGAAAA)1	(GGAA)7	(GAAA)1	(GGAA)2	GGAAAGAATGAA	(GGAA)4	(GGGA)2	102	0
36	(GAGAGGAAGGAAA)1+(GAGAGGAAGGAA)1	(GGAA)9	(GAAA)1	(GGAA)2	GGAAAGAATGAA	(GGAA)4	(GGGA)2	109	0
37	(GAGAGGAAGGAAA)2	(GGAA)6	(GAAA)1	(GGAA)2	GGAAAGAATGAA	(GGAA)1+ (GAAA)1+ (GGAA)2	(GGGA)2	98	0
38	(GAGAGGAAGGAAA)1	(GGAA)10	(GAAA)0	(GGAA)0	GGAAAGAATGAA	(GGAA)4	(GGGA)2	89	0
39	(GAGAGGAAGGAAA)1	(GGAA)5	(GAAA)2	(GGAA)2	GGAAAGAATGAA	(GGAA)4	(GGGA)2	85	0
40	(GAGAGGAAGGAAA)2	(GGAA)2+(GGAAA)1+(GGAA)7	(GAAA)1	(GGAA)2	GGAAAGAATGAA	(GGAA)4	(GGGA)2	115	$5.138x10^{-5}$
**Del**	**x**	**x**	**x**	**x**	**x**	**x**	**x**	**x**	**0.0831**

Supplementary Table 1: Structure, length, and allele frequency in ALSPAC of all READ1 alleles described to date. The six common alleles and the microdeletion are shown in bold.

A.

Phenotype	Description
Reading at 7	Wechsler Objective Reading Dimensions (WORD), single-word reading task, age 7
Reading at 9	Single-word reading task, age 9
Phoneme Del	Auditory Analysis task, age 7
Total IQ	Wechsler Intelligence Scale for Children (WISC), Third Edition, Full-Scale IQ, age 8
Verbal IQ	WISC Verbal IQ component, age 8
Performance IQ	WISC Performance IQ component, age 8
WOLD	Wechsler Objective Language Dimensions (WOLD), verbal comprehension task, age 8
NWR	Non-word repetition task, age 8
NW Read at 9	Non-word reading task, age 9
Spelling at 7	Single-word spelling task, age 7
Spelling at 9	Single-word spelling task, age 9

B.

Phenotype	Description
Severe RD	Cases defined as having a score less than or equal to 2 standard deviations below the mean on the phoneme deletion task
Severe LI	Cases defined as having a score less than or equal to 2 standard deviations below the mean on either the WOLD verbal comprehension task *or* the non-word repetition task

Supplementary Table 2: (A) List of phenotypes used in ALSPAC analyses. Reading measures in the ALSPAC include a phoneme deletion task at age 7, single-word reading at ages 7 and 9, spelling at ages 7 and 9, single non-word reading at age 9, and passage comprehension, speed and accuracy at age 9. The phoneme deletion task measures phoneme awareness, [7] which is widely considered to be a core deficit in RD. [8] For the phoneme deletion task the child listens to a word spoken aloud, and is then asked to remove a specific phoneme from that word to make a new word (e.g. what word is created when the /b/ sound is removed the word 'block'? 'Lock'). This task is also known as the Auditory Analysis Test, and was developed by Rosner and Simon. [9] Single-word reading was assessed at age 7 using the reading subtest of the Wechsler Objective Reading Dimensions (WORD). [10] At age 7 and 9, spelling was assessed; the child was asked to spell a set of 15 age-adjusted words. At age 9, single-word reading was again assessed by asking the child to read ten real words and ten non-words aloud. The words and non-words used are a subset of a larger list of words and non-words taken from research conducted by Terezinha Nunes and others at Oxford. [11] The non-word repetition (NWR) task was ascertained at 8 years of age. This is a verbal language measure wherein the child was asked to repeat recorded non-words. This task measures short-term phonological memory and processing; [12] children with LI consistently perform poorly. [13] Verbal, performance, and total IQ were assessed at age 8, using the Wechsler Intelligence Scale for Children (WISC-III). [14] **(B)** Case/control definitions used in association analysis (Table 1).

Allele	Description	READ1 Alleles	Allele Freq. (ALSPAC)
Clade1	Clusters in Clade 1	5, 6, 11, 12, 13, 14, 20, 21	0.0905
RU1-1	Only 1 copy of RU1	2, 3, 9, 12, 25, 27, 38	0.0521
Short	<90bp	2, 3, 9, 12, 27, 33, 38	0.0521
Long	>102bp	5, 13, 14, 18, 20, 23, 28, 35, 40	0.0400

Supplementary Table 3: Description and allele frequencies for composite READ1 alleles. 'Clade 1' was derived from a ClustalW multiple alignment (standard parameters), and is of interest to us because it contains alleles phylogenetically related to alleles 5 and 6. [3]

	Allele 5	Allele 6	Clade 1	Allele 3	Ru1-1
PD	0.161	0.395	**0.033**	0.379	0.398
Reading7	**0.015**	0.143	**0.004**	0.081	0.135
Reading9	0.569	0.997	0.528	0.542	0.585
NW Reading	0.350	0.506	0.229	0.204	0.169
Spelling7	0.059	0.207	**0.028**	0.097	0.100
Spelling9	0.139	0.292	0.199	0.094	0.146
WOLD	0.549	0.424	0.305	0.955	0.955
NWR	0.423	0.281	**0.038**	0.458	0.191
Total IQ	0.168	0.688	0.059	0.342	0.302
Verbal IQ	0.075	0.273	**0.019**	0.282	0.289
Performance IQ	0.638	0.820	0.448	0.407	0.357

Supplementary Table 4: One-way ANOVA between groups for genetic interactions between READ1 risk and protective alleles and KIAHap (Figure1A-E). Values listed are p-values showing statistical significance of differences between means for the four genotype classes listed in Figure 1, for the indicated phenotype and READ1 single or composite allele. P-values below 0.05 are shown in bold.

**Anchor Primers**	READ1	5'-AGCCCTCCCTACTGACGGAAACACAT-3'<br>5'-TTGCAGGGTGAAAATGAGGAGTTGAAAT-3'
	NRSN1	5'-TGCCCGGTACTCCCTCCAATCAGC-3'<br>5'-CCAAGCCAAGGCCGCAGTGTTC-3'
**Prey Primers**	DCDC2	5'-AGTAAATGGACGCCTGCTGTGT-3'<br>5'-GACTCTTTACTGGGGCTGTTACTATTCTCA-3'
	GPLD1/ALDH5A1	5'-AATATTTTTCTTTCTGCCCCACACC-3'<br>5'-CCCAGCCTCTTCTCCCCCATTTT-3'
	KIA3'	5'-AGCTCCTCCTCCCCTTTCTATTG-3'<br>5'-CATCTGTGGAGGTACGGAGTCTTG-3'
	KIAJ1	5'-TTTATCCTCCCGATTAATTTGTGACATTCC-3'<br>5'-CAGAGCGCCTGGCCGAGAAATA-3'
	KIAJ2	5'-GGGCATTCCTCGCACATCTCATTA-3'<br>5'-CCTCGGCCTGCCAAAGTGCTA-3'
	KIAJ3	5'-TGTCCCATGGTGCTATCAAACC-3'<br>5'-TGCCAGCTGGATTCCAAACA-3'
**Control Primers**	ACTβ	5'-GCCCTAGGCACCAGGGTGTGA-3'<br>5'-ACAGGGTGCTCCTCAGGGGC-3'

Supplementary Table 5: Primer sequences for 3C primers. Primers in black were used to assess fusion fragments for 3C template (anchor + prey). Primers in red are reverse primers with respect to their cognate 3C primers. 3C + reverse primers amplify across the relevant restriction site, and these short amplicons were used with the digested and undigested control template to assess digestion efficiency. Control primers do not amplify across a restriction site; they generate a short amplicon from the *ACTβ* gene, which was used to normalize across different qPCR templates.

**Anchor Primer**	Globin_C	5'-CGGTCATCCTCACGGTGACTAACGCA-3'
**Prey Primers**	Globin_A	5'-GACTCTTGAGGGCCTGACCTCGCTTAC-3'
	Globin_B	5'-GGTGGCAAAAGGCCTGTGCTGTTAGA-3'
	Globin_D	5'-AATGGCAATCACCACGATGGCCACA-3'

Supplementary Table 6: Primer sequences for control 3C experiment at the β-globin locus. [6]

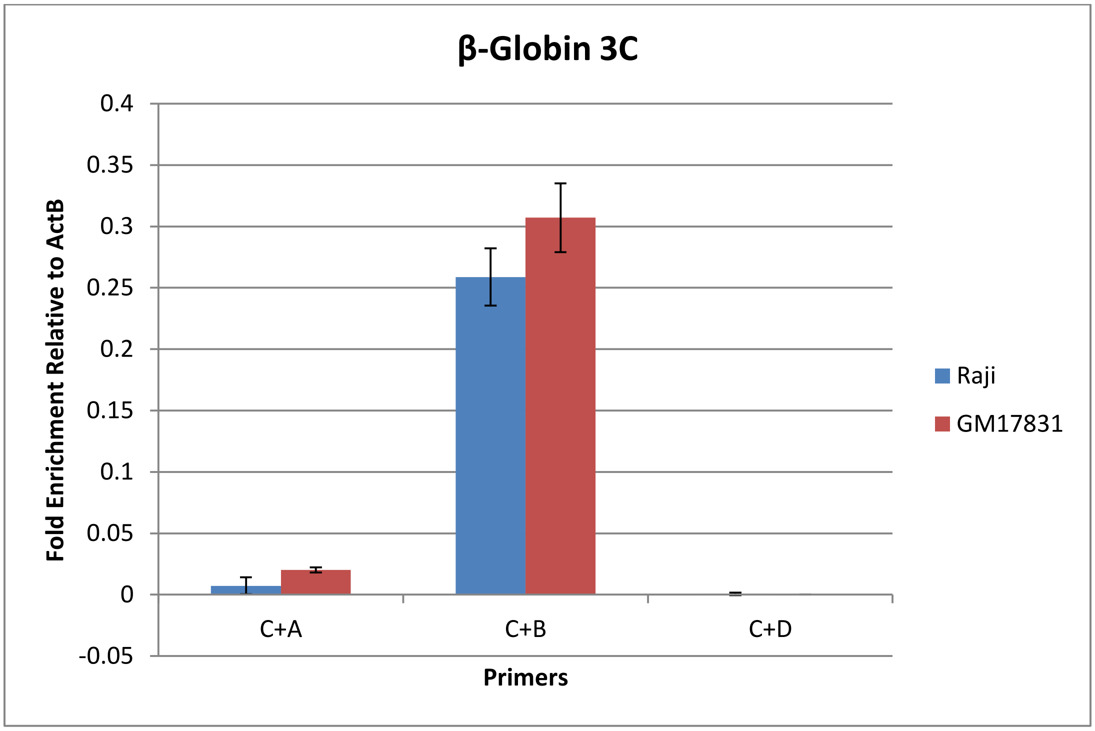

Supplementary Figure 1: Results of the β-globin control 3C experiment. The y-axis shows fold-enrichment of the indicated fusion fragment relative to the control *ACTβ* primers, which were used to normalize across 3C templates. Error bars represent standard error among three replicates. These results agree with previously reported findings for this locus, [6] and indicate an effective 3C protocol.

Supplementary References

1. Boyd A, Golding J, Macleod J, Lawlor DA, Fraser A, Henderson J, Molloy L, Ness A, Ring S, Davey Smith G. Cohort Profile: The 'Children of the 90s'--the index offspring of the Avon Longitudinal Study of Parents and Children. International journal of epidemiology 2012;**42**(1):111-27 doi: 10.1093/ije/dys064[published Online First: Epub Date]|.
2. Gayan J, Smith SD, Cherny SS, Cardon LR, Fulker DW, Brower AM, Olson RK, Pennington BF, DeFries JC. Quantitative-trait locus for specific language and reading deficits on chromosome 6p. American journal of human genetics 1999;**64**(1):157-64 doi: 10.1086/302191[published Online First: Epub Date]|.
3. Powers NR, Eicher JD, Butter F, Kong Y, Miller LL, Ring SM, Mann M, Gruen JR. Alleles of a Polymorphic ETV6 Binding Site in DCDC2 Confer Risk of Reading and Language Impairment. American journal of human genetics 2013;**93**(1):19-28 doi: 10.1016/j.ajhg.2013.05.008[published Online First: Epub Date]|.
4. Scerri TS, Morris AP, Buckingham LL, Newbury DF, Miller LL, Monaco AP, Bishop DV, Paracchini S. DCDC2, KIAA0319 and CMIP are associated with reading-related traits. Biological psychiatry 2011;**70**(3):237-45 doi: 10.1016/j.biopsych.2011.02.005[published Online First: Epub Date]|.
5. Miele A, Dekker J. Mapping cis- and trans- chromatin interaction networks using chromosome conformation capture (3C). Methods Mol Biol 2009;**464**:105-21 doi: 10.1007/978-1-60327-461-6_7[published Online First: Epub Date]|.
6. Vu TH, Nguyen AH, Hoffman AR. Loss of IGF2 imprinting is associated with abrogation of long-range intrachromosomal interactions in human cancer cells. Human molecular genetics 2010;**19**(5):901-19 doi: 10.1093/hmg/ddp558[published Online First: Epub Date]|.
7. Hulme C, Goetz K, Gooch D, Adams J, Snowling MJ. Paired-associate learning, phoneme awareness, and learning to read. Journal of experimental child psychology 2007;**96**(2):150-66 doi: 10.1016/j.jecp.2006.09.002[published Online First: Epub Date]|.
8. Peterson RL, Pennington BF. Developmental dyslexia. Lancet 2012;**379**(9830):1997-2007 doi: 10.1016/S0140-6736(12)60198-6[published Online First: Epub Date]|.
9. Rosner J, Simon DP. The Auditory Analysis Test: An Initial Report. Journal of Learning Disabilities 1971;**4**(384):40-48 doi: 10.1177/002221947100400706[published Online First: Epub Date]|.
10. Rust J, Golombok S, Trickey G. *WORD: Wechsler Objective Reading Dimensions Manual*. Sidcup, UK: Psychological Corporation, 1993.
11. Nunes T, Bryant P, Olssen J. Learning Morphological and Phonological Spelling Rules: An Intervention Study. Scientific Studies of Reading 2003;**7**(3):289-307 doi: 10.1207/S1532799XSSR0703_6[published Online First: Epub Date]|.
12. Gathercole SE, Baddeley AD. *The Children's Test of Nonword Repetition*. London, UK: Psychological Corporation, 1996.
13. Bishop DV, North T, Donlan C. Nonword repetition as a behavioural marker for inherited language impairment: evidence from a twin study. Journal of child psychology and psychiatry, and allied disciplines 1996;**37**(4):391-403
14. Wechsler D. *Wechsler Intelligence Scale for Children, Fourth Edition*. London, UK: The Psychological Corporation, 2004.
